# Exposure to Folate Receptor Alpha Antibodies during Gestation and Weaning Leads to Severe Behavioral Deficits in Rats: A Pilot Study

**DOI:** 10.1371/journal.pone.0152249

**Published:** 2016-03-24

**Authors:** Jeffrey M. Sequeira, Ankuri Desai, Maria I. Berrocal-Zaragoza, Michelle M. Murphy, Joan D. Fernandez-Ballart, Edward V. Quadros

**Affiliations:** 1 Departments of Medicine, State University New York (SUNY)-Downstate Medical Center, Brooklyn, New York, 11209, United States of America; 2 The School of Graduate Studies, State University New York (SUNY)-Downstate Medical Center, Brooklyn, New York, 11209, United States of America; 3 Preventive Medicine and Public Health and IISPV Faculty of Medicine and Health Sciences, Universitat Rovira i Virgili, Reus, Tarragona, Spain; Université Pierre et Marie Curie, FRANCE

## Abstract

The central nervous system continues to develop during gestation and after birth, and folate is an essential nutrient in this process. Folate deficiency and folate receptor alpha autoantibodies (FRα-AuAb) have been associated with pregnancy-related complications and neurodevelopmental disorders. In this pilot study, we investigated the effect of exposure to FRα antibodies (Ab) during gestation (GST), the pre-weaning (PRW), and the post weaning (POW) periods on learning and behavior in adulthood in a rat model. In the open field test and novel object recognition task, which examine locomotor activity and anxiety-like behavior, deficits in rats exposed to Ab during gestation and pre-weaning (GST+PRW) included more time spent in the periphery or corner areas, less time in the central area, frequent self-grooming akin to stereotypy, and longer time to explore a novel object compared to a control group; these are all indicative of increased levels of anxiety. In the place avoidance tasks that assess learning and spatial memory formation, only 30% of GST+PRW rats were able to learn the passive place avoidance task. None of these rats learned the active place avoidance task indicating severe learning deficits and cognitive impairment. Similar but less severe deficits were observed in rats exposed to Ab during GST alone or only during the PRW period, suggesting the extreme sensitivity of the fetal as well as the neonatal rat brain to the deleterious effects of exposure to Ab during this period. Behavioral deficits were not seen in rats exposed to antibody post weaning. These observations have implications in the pathology of FRα-AuAb associated with neural tube defect pregnancy, preterm birth and neurodevelopmental disorders including autism.

## Introduction

Folate deficiency in humans leads to megaloblastic anemia and in women of child bearing age, could lead to difficulty in conceiving, miscarriage, and neural tube defects (NTD) in the fetus and preterm birth [1, 2]. The strongest evidence supporting the beneficial effects of folate in pregnancy has come from prenatal folic acid supplementation that has dramatically reduced the incidence of NTD pregnancies [3]. Animal models of folate deficiency in mice and rats have shown developmental and behavioral deficits in the offspring [4, 5]. In the absence of severe deficiency, suboptimal folate status could lead to subtle structural changes in the brain that could produce functional deficits in later life. Evidence in support of this conclusion is provided by animal models on folate-restricted diets [6, 7]. In the absence of dietary deficiency, genetic and metabolic defects could also disrupt folate utilization [8, 9]. Another mechanism by which folate metabolism could be disrupted is an autoimmune disorder whereby an autoantibody to the FRα could interfere by blocking folate uptake and by triggering an immune reaction involving inflammation. Such autoantibodies have been reported in women with a history of NTD pregnancy [10], Rett syndrome [11], and low functioning autism [12]. Despite the association of FRα-AuAb with numerous developmental disorders, proof that antibodies directed against the FRα could affect brain development and function is lacking. Exposing a pregnant rat to placental folate receptor (a mixture of antibodies to FRα and FRβ) antiserum on gestational day 8 (GD8) has previously shown to produce developmental anomalies, or complete resorption of embryonic implants depending on the dose of the antiserum administered [13]. The gross malformations observed led to the conclusion that FRAuAb could contribute to the pathology of NTD and cranio-facial abnormalities. Some studies have associated the presence of these antibodies with NTD pregnancy [10, 14, 15], while others did not find a significant association with NTD pregnancy [16, 17]. The presence of these FRα-AuAb has been associated with cerebral folate deficiency (CFD) in children [18]. This neurological syndrome develops 4–6 months after birth and is characterized by low cerebrospinal fluid (CSF) folate concentration despite adequate plasma folate [19]. Recently, two independent studies have reported the association of FRα-AuAb with autism spectrum disorders [20, 21].

While the teratogenic effects of FRα-Ab have been reported [13], the effect of exposure to a lower dose of FRα-Ab during fetal and neonatal brain development is not known. The role of folate in neurodevelopment during embryogenesis is well established [22, 23]. After birth, brain development continues with functional refinements and folate may be an essential nutrient in this process since folate deficiency in the pups during the pre weaning period results in behavioral deficits in adulthood [5]. Exposure to low doses of FRα-Ab during critical periods of neuronal development could affect the structural and functional refinement of the developing brain. The aim of the present study was to determine the effect of exposure to FRα-Ab, during GST, PRW and the POW periods on behavior, learning and memory functions in the rat.

## Material and Methods

### Production of recombinant folate receptor

The rat folate receptor alpha cDNA [24] corresponding to AA1-225 was amplified by PCR and cloned into plasmid pcDNA 3.1. HEK293 cells were transfected with the plasmid containing the truncated cDNA. Stable clones expressing the secreted form of the FRα were cultured in geneticin-containing medium. The protein was purified by affinity chromatography on a folic acid -Sepharose matrix as previously described [25].

### Generation of polyclonal antiserum to folate receptor

To produce antiserum, 2 Wistar white rabbits were injected subcutaneously with 200μg of purified rat FRα antigen in complete Freund’s adjuvant followed by 3 injections of the antigen in incomplete adjuvant over a 3-month period. The rabbits were bled and tested for antibody 2 weeks after the last injection. Repeated bleeds were obtained every week for 4 weeks followed by boosting with antigen. This was done for 3 cycles to obtain a large amount of high titer antiserum. The antiserum contained a mixture of 2 types of antibodies: binding and blocking antibodies to rat FRα. The IgG fraction in the antiserum was purified by affinity chromatography on a protein A matrix, the titer determined and used in the study.

#### Binding antibody titer

**Fig 1:**
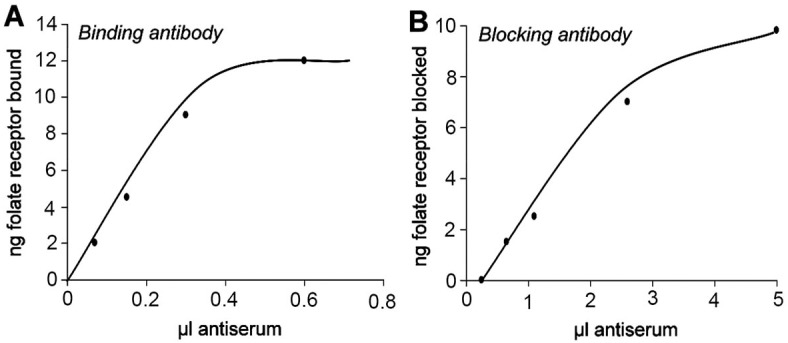
Anti FRα antibody titer. Binding (A) and blocking (B) titer of rabbit polyclonal antibody to recombinant rat folate receptor alpha.

Binding antibody is defined as an antibody that binds to an epitope distant from the folate binding site and does not interfere with folate binding to FRα. Purified FRα was diluted in buffer to bind ~10000 cpm ^3^H-pteroylglutamic acid (^3^H-PGA, Moravek Inc.). The protein was incubated with the radio-labelled tracer for 20 minutes followed by addition of 100μl of diluted antiserum and incubation at 4°C overnight. The FRα-antibody complex was captured on protein A membranes (Calbiochem) and radioactivity measured (**Fig 1A**).

#### Blocking antibody titer

Blocking antibody is defined as an antibody that prevents the binding of folate when preincubated with apo-FRα by virtue of the antibody directly or sterically interfering with folate binding. Purified FRα was diluted to bind ~10000 cpm ^3^H-PGA and incubated at 4°C overnight with 100μl of diluted antiserum. Next day, 20000 cpm ^3^H-PGA was added to the reaction and incubated at room temperature for 20 minutes. Free ^3^H-PGA was separated from protein bound ^3^H-PGA with dextran-coated charcoal. Blocking activity of the antiserum was expressed as nanograms of FRα blocked from binding ^3^H-PGA (**Fig 1B).**

### Study design

Timed-pregnant Long Evans hooded rats (Charles River Laboratories International, Inc. Wilmington, MA) were anesthetized on GD8 and a laparotomy was performed to record the number of implanted embryos before the administration of FRα-Ab. Rats undergoing a laparotomy were anesthetized with ketamine:xylazine (80 mg/kg: 10mg/kg IP). Under aseptic conditions, a 1.5 inch midline incision was made, the embryonic implants were counted and the incision closed with two layers of # 4–0 silk sutures. For any post-procedural pain, relief was provided by administering buprenorphine (0.05 mg/kg SC) at the time of the procedure and then twice daily for one week post-surgery. Normal rat serum was used as a carrier protein along with the antibody to prevent proteolytic degradation of the antibody in the peritoneal cavity. The FRα-Ab at a dose of 4μg / embryo, mixed with 1ml normal rat serum was administered by intra-peritoneal (IP) injection one hour after the laparotomy. This dose was not lethal to the embryos and permitted the implanted embryos to be carried to term. Pups born received additional FRα-Ab (35μg per pup per day) on post-natal days (PND) 10, 11 and 12 (GST+PRW group). Another pregnant dam received a single dose of antibody at 4.0 μg / embryo on GD8 and her pups did not receive any additional doses (GST group). An additional dam did not receive any antibody during gestation but the pups born received antibody (35μg in 0.2ml rat serum) on PND 10, 11 and 12 (PRW group). An additional dam did not receive any antibody during gestation but the pups born received antibody (50μg in 0.2ml rat serum IP) on PND 26, 27 and 28 (POW group). Pups from all of the above groups underwent various behavioral tests (open field test, novel object recognition task and place avoidance tasks) between PND40 and PND70. We used a control group of dams and pups that did not undergo surgery and were never exposed to FRα-Ab or to an equivalent dose of normal rabbit IgG, to determine normal parameters of behavioral tests. Sham-control (SC) dams underwent identical surgery with anesthesia, but received normal rabbit IgG in rat serum, rather than the anti FRα-IgG Ab in rat serum. The SC dam and pups appeared and behaved identically to controls tested previously (rats that have not undergone any surgery or exposure to vehicle). Consequently, a separate control group to evaluate the effect of normal rat serum was not included as the SC rats did not behave differently from untreated controls which suggests that vehicle (as well as normal rabbit IgG) had no effect on parameters tested. All rats were fed a normal diet containing 2mg folic acid / kg chow as recommended by the American Institute of Nutrition (1977) and they had *ad libitum* access to food and water throughout the study. They were maintained at 22°C and a 12 hour light / dark cycle. All efforts were made to minimize suffering. The experimental protocols were approved by the Animal Care and Use Committee of the State University of New York, Downstate Medical Center.

### Behavioral tests

We used the open field test to assess anxiety-induced locomotor activity and exploratory behavior [26]. Rat movement and behavior were recorded for 5-minute sessions in the open field (60cm wide x 60cm deep with 40cm high walls and the floor was divided into a grid of 5x5 cm squares). The open field arena was cleaned with 30% ethanol between tests to prevent olfactory cues from influencing the behavior of other rats. The parameters analysed by an observer blinded to the experimental conditions included distance travelled (cm) and time (sec) spent in peripheral, central and corner areas of the field, rearing behavior and number of self-groomings.

Rodents spend much more time exploring a novel object when introduced to it in the presence of a familiar object. Visual recognition and behavior are intrinsically linked. The novel object recognition test [27] assesses the animal’s recognition memory. This test consists of three phases [28]. In the first phase the rat was habituated to the environment by being placed in the arena (same size as the open field test apparatus described previously) for 30 minutes each on two consecutive days. In the second phase of familiarization, two identical objects were placed in the habituation arena. The time that the rat spent exploring each object during a 5-minute session was recorded. “Exploration” refers to the time that the rat spent with its head facing and within two centimetres of the object, sniffing, licking and biting it. After this timed session, one of the explored objects was switched with a novel object and the time spent exploring familiar and novel objects in a 5 minute session was recorded and analyzed.

The place avoidance tasks require recognition and segregation of information obtained from both relevant and irrelevant stimulus sources and permits the assessment of spatial memory formation [29–32]. The apparatus used in this test consisted of a rotating stainless steel arena situated in a room that contains visual cues on the walls. The animals have to use these cues to localize a shock zone in the arena. The test consists of 3 phases conducted over 3 consecutive days: passive, active and conflict place avoidance tasks. A 60° invisible stationary shock zone is defined within the round arena. When the rat enters the shock zone it receives an aversive foot shock (0.2 mA, 60 Hz, 500 ms) every 1.5 s until the animal leaves the zone. The passive place avoidance task consists of 4 trials of 10 minutes each with 10 minutes rest period in between each trial. For this phase of the test the animal can use olfactory (or arena) and visual (or room) cues to learn where the shock zone is and how to avoid it. Olfactory cues from feces, urine and scent marks deposited in the arena during the trial and visual cues of different marks or objects placed on the walls surrounding the apparatus can be used. The next day, in the active place avoidance task, the shock zone remains in the same coordinates as the previous day, but the arena is rotated at 1 rpm. For this test the rat must ignore olfactory spatial cues and focus on the visual wall marks in the room to avoid the shock zone. This phase determines the rats’ ability to avoid the shock sector in a rotating arena by segregating stationary visual cues from the rotating local olfactory cues. The rats are given up to 6 trials to master this task. On the third day, in the first trial the rat is tested for active place avoidance to determine if long-term memory is intact and the rat is able to recollect what was learnt the previous day. Following this, the shock zone is flipped 180° for the conflict place avoidance task whereby the rat has to suppress old memory and learn a new task. A number of parameters are recorded by an automated computer acquisition system (Bio-Signal Group Corp., New York, NY, USA). During each trial the distance moved (in meters), the number of entrances into the shock zone, the number of entrances per distance moved (1/m), the maximum avoidance time (s) and distance travelled (m) to do so, the total number of shocks received per trial and delay prior to first entry into the shock sector were recorded. Learning is defined as a process during the different trials in which rats reduced the number of entrances into the shock zone during each trial and increased time to first entry over the subsequent trials.

### Statistical analysis

No sex difference was observed for any of the parameters tested and therefore data from both males and females was pooled for each group. We compared the results of the different variables of the open field and novel object recognition tests between GST+PRW exposed and SC groups using the Mann-Whitney U test because the data were not normally distributed, and did a cross-sectional and a longitudinal analysis of the place avoidance tasks. We used the one tailed test because the expected direction of the effect of treatment with FRα-Ab was uni-directional and based on previous observations that folate deficiency and exposure to FRα antibody are associated with neurodevelopment disorders in rats. The cross-sectional analysis consisted of comparing the results of the parameters in each trial of the passive, active and conflict place avoidance tasks between FRα-Ab exposed and SC groups. Each group was studied longitudinally in the different phases of place avoidance tasks using the Wilcoxon signed-rank test. We did two types of comparisons in the place avoidance tasks: a) first trial with the rest of trials (trial 1-trial 2, trial 1-trial 3 and trial 1-trial 4) and b) each trial with the previous one (trial 2-trial 1, trial 3-trial 2 and trial 4-trial 3). Bonferroni correction was applied to the P values to account for increased alpha risk due to multiple comparisons. The significance level for all tests was set at p<0.05. Statistical analysis was performed using the Statistical Package for the Social Sciences, version 17.0 for Windows (SPSS Inc., Chicago, IL, USA).

## Results

**Fig 2:**
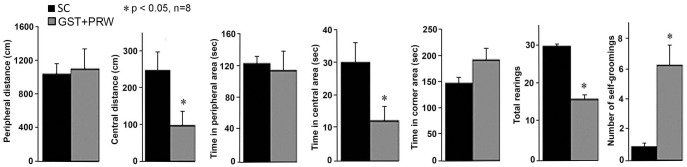
Results of the open field test. Summary data of the various parameters evaluated in the open field test for rats exposed to FRα antibody during GD 8 and PND 10–12 (GST+PRW group). Antibody exposed rats covered less distance and spent less time in the central area, had fewer rearings and significantly increased number of self-grooming. (Mean + SEM; *P<0,05 compared to SC group).

At a dose of 4μg per embryo, all embryos were carried to term and the pups born received 35ug of FRα antibody in 0.2 ml rat serum on PND 10, 11 and 12 (GST+PRW group). When these rats were tested for behavior between PND 40–70 they showed no differences in the total distance traveled in the open field test compared to SC group. However, the GST+PRW group (n = 6) spent less time in the center of the open field compared with SC group (n = 13) (median for GST+PRW group *vs* SC group: 13 *vs* 30 sec; U = 20.5, p = 0.052), covered a shorter distance in the central area (102 *vs* 252cm; U = 16.0, p = 0.021), spent more time in the corner (185 *vs* 149sec; U = 20.0, p = 0.048) and had an increased number of self-groomings (6 *vs* 1; U = 0.0, p<0.001) (Fig 2). In the novel object recognition test, compared to the SC group (n = 6), the GST+PRW group (n = 6) spent less time exploring the novel object. These rats approached both objects fewer times and spent less time with both objects than the SC group although the difference was not significant. The GST+PRW group also took longer to start exploring objects than the SC group [median (n): 35.5 (6) *vs* 8.0s (6), respectively; U = 5.0, p = 0.018].

**Fig 3:**
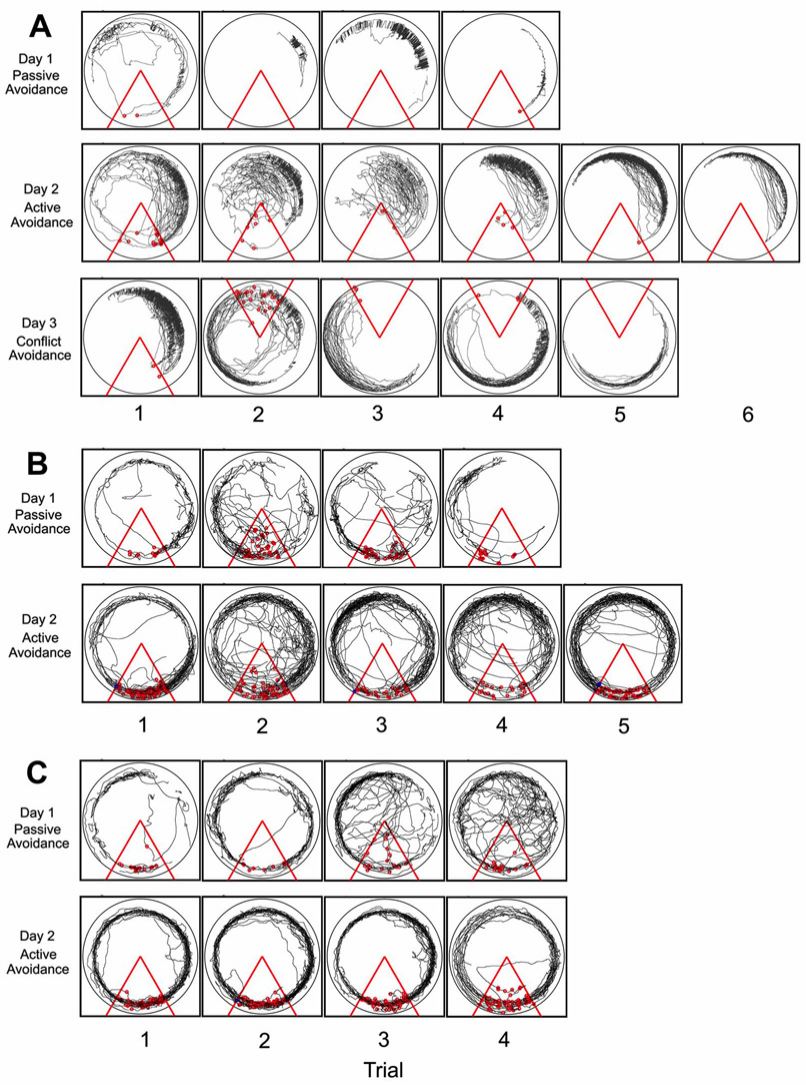
Tracking the movement of the rat in the place avoidance arena. Tracings of the rat’s movement in the place avoidance arena for a SC rat (A), a GST+PRW rat able to learn the passive place avoidance task but fails the active place avoidance task (B) and a GST+PRW rat unable to learn both passive and active place avoidance tasks (C). The dots in the triangle represent number of shocks received during that trial.

Representative tracings of the movement of the rat in the arena along with the shocks received during the learning process of the various place avoidance tasks is shown in Fig 3. While a normal rat and the SC rat is able to learn all of the tasks in 4–6 trials, the affected rats (GST+PRW) were unable to learn these tasks as seen by the inability of the rat to avoid the shock zone.

**Fig 4:**
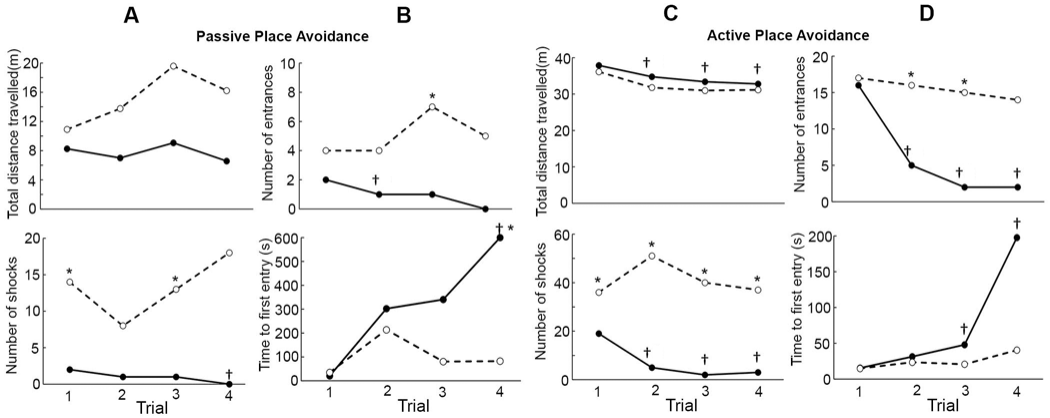
Place avoidance parameters in SC and GST-PRW rats. Passive and active place avoidance parameters in different trials for normal rabbit IgG exposed rats (-●-; SC group; *n* = 8) and GST-PRW rats exposed to FRα Ab (-○-; GST-PRW group; *n* = 6). Inability of the GST-PRW rats to learn the passive place avoidance (Panels A and B) and the active place avoidance (Panels C and D) is evident from the lack of a decrease in number of entrances and the shocks received along with lack of a decrease in time to first entry in subsequent trials. Median values for each parameter are reported. *Different from the SC group (*P* < 0.05). †The first trial (in the control group) was different from the rest of the trials (*P* < 0.05). Bonferroni correction was applied to the *P* values for multiple comparisons.

The GST+PRW group had difficulty in performing the passive place avoidance task with 70% of the rats unable to complete the task. This group covered similar distance in all the trials compared to the SC group. The number of times the GST+PRW group entered the shock zone was higher and they did not reduce the number of entrances in subsequent trials compared to the SC group. The GST+PRW group had difficulty in avoiding the shock zone and they received more shocks than SC over the same time interval. In the SC group, an increase in the time to first entry into the shock area was observed with subsequent trials. This is an indication that the SC animal remembers the location of the zone from the previous trial. In the last two trials, the SC rats delayed entering the shock zone for longer than the GST+PRW group in each trial and in successive trials. None of the rats in the GST+PRW group learned the active place avoidance task as indicated by a lack of improvement in most of the parameters recorded (Fig 4). They entered the shock area more frequently, received more shocks, had more entrances per distance covered and moved more to avoid the shock zone and entered the shock zone more frequently compared to the SC group. During the active place avoidance task three rats in the GST+PRW group remained completely still for up to 2 minutes in the rotating arena. They did not show any observable body movement and this “freezing” behavior continued even into the shock zone.

**Fig 5:**
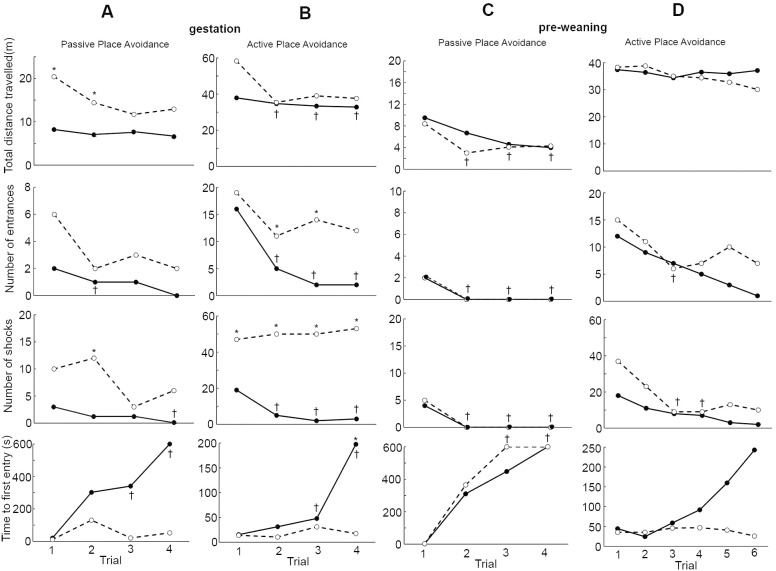
Place avoidance in GST, PRW and SC rats. Parameters for the place avoidance tasks for GST (-○-, n = 4) and SC (-●-, n = 8) rats (Panels A and B) or PRW (-○-, n = 8) and SC (-●-, n = 4) rats (Panels C and D). Median values for each parameter are reported. *Different from the control group (*P* < 0.05); ^†^different from the first trial (*P* < 0.05). Note: in Panel C, ^†^ indicates difference compared to the first trial in the exposed group (*P* < 0.05). Bonferroni correction for multiple comparisons was applied to the *P* values.

The severe functional deficits seen in the GST+PRW rats prompted us to test exposure to antibody during gestation (GST) and pre-weaning (PRW) separately. The GST rats were able to learn the passive place avoidance task, as seen by a decrease in the number of entrances with subsequent trials, parallel to the decrease seen in SC animals. However, unlike the SC group, time to first entry did not increase with subsequent trials in the GST group. GST rats could not learn the active avoidance task since they had a significantly higher number of entrances into the shock zone, received a greater number of shocks and could not decrease the number of entrances into the shock zone in subsequent trials like the SC rats (Fig 5, panels A and B). Similar but somewhat less severe learning deficits were seen in PRW rats. Like the GST rats, the PRW rats were able to learn the passive place avoidance task and perform much better in the active place avoidance task (Fig 5, panels C and D). The only deficit seen in these rats was their inability to increase time to first entry in the active place avoidance task. Both GST only and PRW only groups could not learn the conflict place avoidance task.

**Fig 6:**
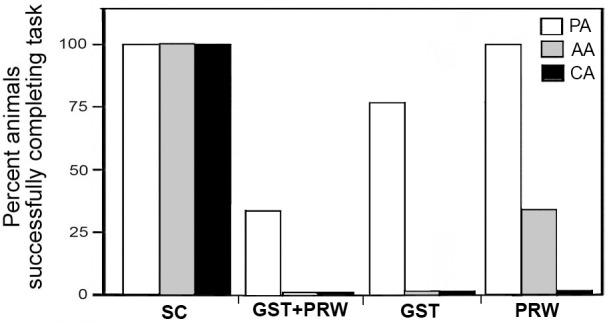
Summary data of the place avoidance tasks for SC, GST+PRW, GST and PRW rats. While 30% of the GST+PRW rats learned the passive place avoidance task (PA), none were able to learn the active place avoidance task (AA). As per protocol, since all the GST+PRW rats failed the active place avoidance task, they were not tested for the conflict place avoidance task (CA). Similar but less severe effects were seen in GST only and PRW only rats.

Overall, 30% of the GST+PRW rats learned the passive place avoidance task but all failed to learn the active place avoidance task (Fig 6). Rats exposed to antibody only during gestation or preweaning, overall were less severely affected with deficits similar to those seen in GST+PRW rats when tested in the open field (S1 Fig). Additionally, in the place avoidance tests, 75% of the GST rats and 100% of the PRW rats were able to complete the passive place avoidance task and 0% and 38% were able to learn the active place avoidance task respectively. However, none succeeded in the conflict place avoidance (Fig 6).

In contrast, pups exposed to antibody after the weaning period (POW) on post-natal days 26 to 28 performed similarly to the SC rats in the open field test (S2 Fig) and successfully completed the place avoidance tasks, behaving similarly to SC rats (S3 Fig).

## Discussion

Deficits noted in the open field and novel object tests, including excessive grooming in GST+PRW rats indicates the presence of an anxiety-related disorder. These are important behavioral measurements in rodent experimental anxiety models [33]. Although the GST+PRW rats were interested in exploring both objects and preferred the novel to the familiar object when shown together, they were overtly cautious and hesitant about initiating exploration. Learning and memory deficits in these GST+PRW rats were evident from their inability to learn the passive place avoidance task with only 30% of the animals able to learn to avoid a defined shock zone in a stationary arena. Learning deficits were evident from their inability to increase time to first entry in subsequent trials and decrease the number of shocks received.

In the active place avoidance test a different strategy to that used in the passive avoidance is needed to respond to the new conditions of the task [29]. Olfactory stimuli are neutralized by the rotation of the arena and therefore the rat can only use visual clues outside of the arena to help in avoiding the shock zone. All of the GST+PRW rats failed to learn the active place avoidance task. They also failed to decrease the number of entries into the shock zone and number of shocks received with subsequent trials. Since there was no significant difference in the total distance travelled in the arena between GST+PRW and SC rats, motor function was not compromised in GST+PRW rats.

The place avoidance task uses an aversive stimulus for the rat to learn a task. The foot shock intensity used (0.2 mA) is not sufficient to cause a fearful reaction in healthy rats. Three out of six GST+PRW rats showed freezing behavior in some trials of the active avoidance task. Basolateral amygdala and hippocampus [34, 35] and some neurotransmitters such as serotonin [36] have been shown to be associated with this freezing behavior. The dose and window(s) of exposure to the antibody appear to be critical determinants of the pathology since rats exposed to antibody *in utero* during GD 8–10 (GST) or during PND 10–12 (PRW) alone were less severely affected and showed marked improvement over animals exposed to antibody both during gestation and weaning (GST+PRW), which show severe learning and memory deficits. FRα-Ab exposure after weaning during PND 26–28 (POW) had no effect on learning and behavior in the rats. This attests to the extreme susceptibility of the fetal and neonatal brain to the pathologic effects of the antibody. This could also explain why women who develop autoantibodies to FRα are not affected but could render the fetus at risk for developmental disorders. This is the first pilot study demonstrating the effects of exposure to FRα specific antibodies during gestation and weaning on behavior, learning and memory in later life. The time frames of gestation and the pre weaning periods chosen correspond respectively to fetal development and the neonatal period in humans [37]. During this time, the brain could be highly susceptible to folate depletion, inflammation and oxidative stress. The limitation of this pilot study is that all of the pups for each of the groups were from a single dam. Nevertheless, the significant effect observed in GST+PRW, GST and PRW and lack of any effect in the POW is strong evidence of the pathologic effect and importance of timing of exposure to FRα antibodies during development. Many of the human developmental disorders have their origins *in utero* with maternal FRα autoantibodies posing a potential risk factor contributing to the pathology [21]. During fetal life, free movement of maternal IgG antibodies could seek their target antigen in the fetus. These same antibodies could also block transplacental transport and fetal uptake of folate. Some of the neurologic disorders associated with FRα autoantibodies develop in early infancy. These children appear normal at birth but start deteriorating developmentally around 6–12 months of age. They appear to develop FRα autoantibodies around this same time when they are switched from breast-feeding to bovine milk. Most of these children also have gastro-intestinal disturbances and the FRα in the milk appears to raise the autoantibody titer in these children [38]. A milk-free diet and high dose folinic acid has proven to be beneficial in the treatment of these children. The presentation of the FRα autoimmune disorder is also very similar in autism and pharmacologic doses of folinic acid (1–2mg/kg per day; max 50mg) has shown remarkable improvements in the core symptoms of autism spectrum disorders [20, 21]. The neurologic deficits in autism can vary widely with multiple underlying causes with multiple pathways and genes involved. The FRα autoimmune disorder and folate deficiency could contribute to the diversity of the pathology by affecting imprinting genes and epigenetic effects on expression of many of the genes involved. With the spectre of autism on the rise, primarily from increased awareness and improved diagnosis, clinical trials with adequate folinic acid supplementation of women of child-bearing age, specifically those positive for the folate receptor autoantibody is warranted. Breast feeding for longer periods, identifying children positive for the FRα autoantibody and treating them early with folinic acid may prevent many of the neurologic deficits.

## Supporting Information

S1 Dataset Fig 2 Data. (XLS)

S2 Dataset Fig 4 Data. (XLSX)

S3 Dataset Fig 5 Data. (XLSX)

S4 Dataset S1 Fig Data. (XLSX)

S5 Dataset S2 Fig Data. (XLSX)

S6 Dataset S3 Fig Data. (XLSX)

S1 Fig Open Field Testing of GST (A) and PRW (B) rats. Rats exposed to FRα-Ab during the GST (GD 8, n = 4; SC, n = 10) and PRW (PND 10–12) period (n = 9; SC n = 5) showed some deficits in the open field test compared to SC. GST rats had significantly fewer rearings compared to the SC. PRW rats spent significantly increased time in the corner areas and significantly decreased time in the central area. These are indicative of anxiety-like behaviour (*p<0.05).
(JPG)

S2 Fig Open Field Testing for POW: Rats exposed to FRα-Ab during the POW period (PND 26–28) behaved similarly to SC in the open field test, with no significant differences noted. (JPG)

S3 Fig Place Avoidance Testing in POW rats. Rats exposed to FRα-Ab during the POW period successfully completed the active and conflict place avoidance tasks similarly to SC rats. They showed similar distance travelled as well as a decrease in entrances in subsequent trials, indicating successful learning of the task.
(JPG)
