# Supplementary figures and images for: Exposure to Folate Receptor Alpha Antibodies during Gestation and Weaning Leads to Severe Behavioral Deficits in Rats: A Pilot Study
Source: PLoS One. 2016 Mar 24;11(3):e0152249. doi: 10.1371/journal.pone.0152249 (PMC4807076; doi:10.1371/journal.pone.0152249)

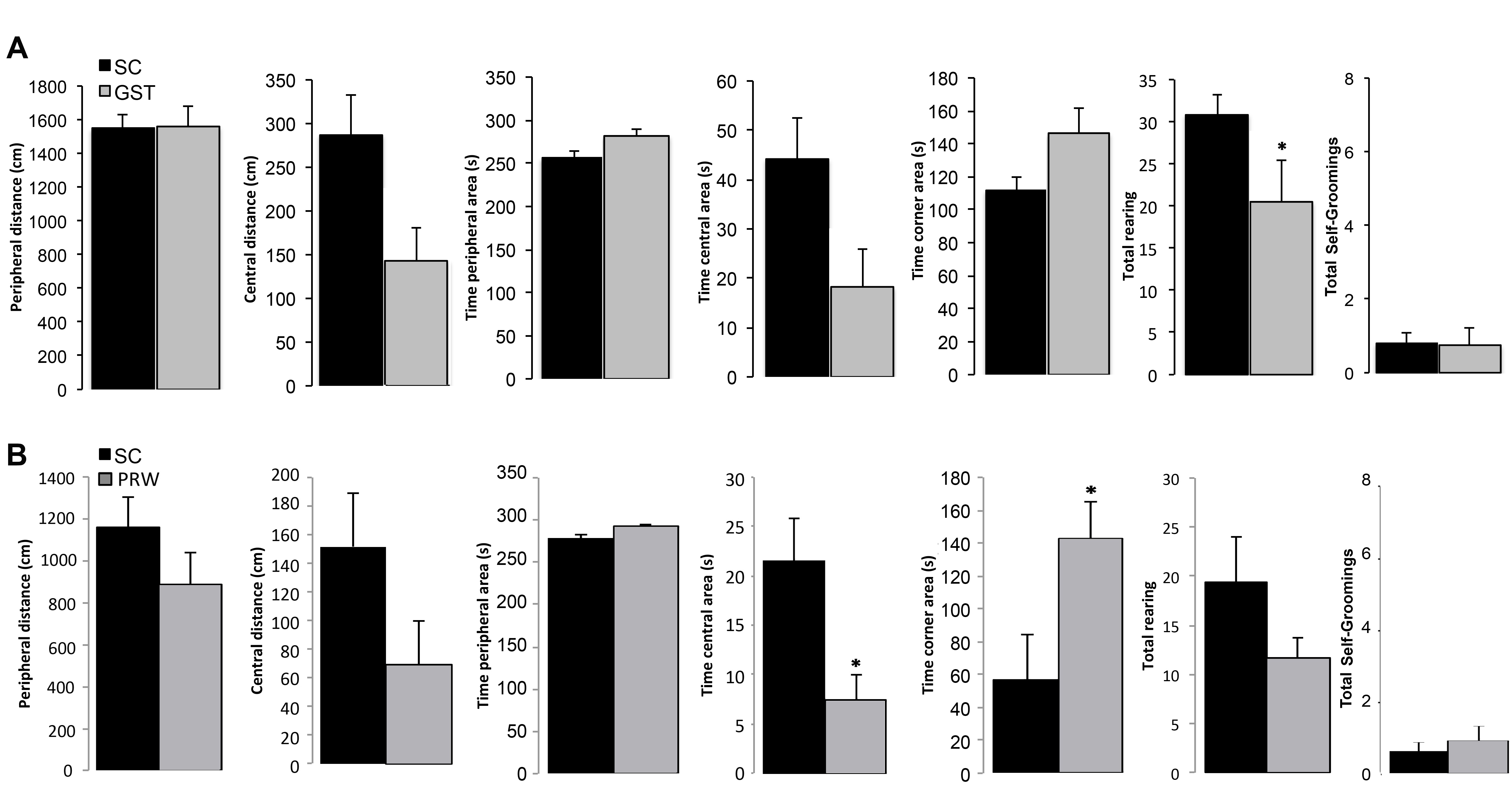

Supplement: S1 Fig — Open Field Testing of GST (A) and PRW (B) rats. Rats exposed to FRα-Ab during the GST (GD 8, n = 4; SC, n = 10) and PRW (PND 10–12) period (n = 9; SC n = 5) showed some deficits in the open field test compared to SC. GST rats had significantly fewer rearings compared to the SC. PRW rats spent significantly increased time in the corner areas and significantly decreased time in the central area. These are indicative of anxiety-like behaviour (*p<0.05). (JPG) [file pone.0152249.s007.jpg]

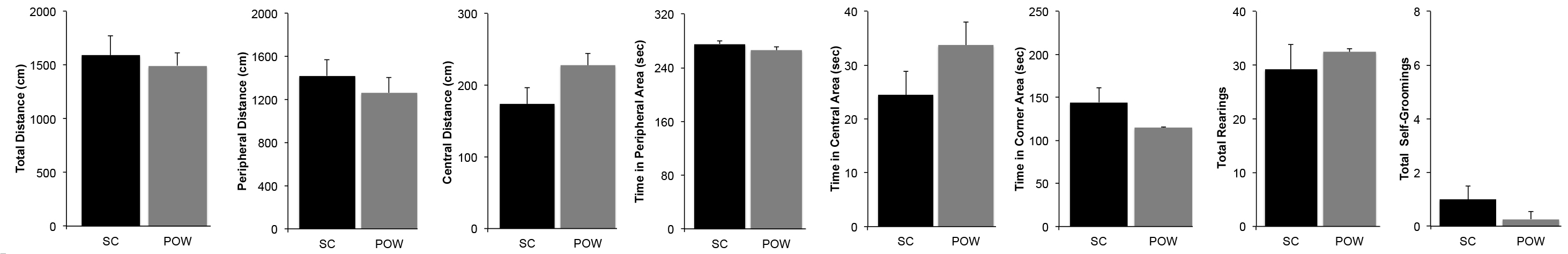

Supplement: S2 Fig — (JPG) [file pone.0152249.s008.jpg]

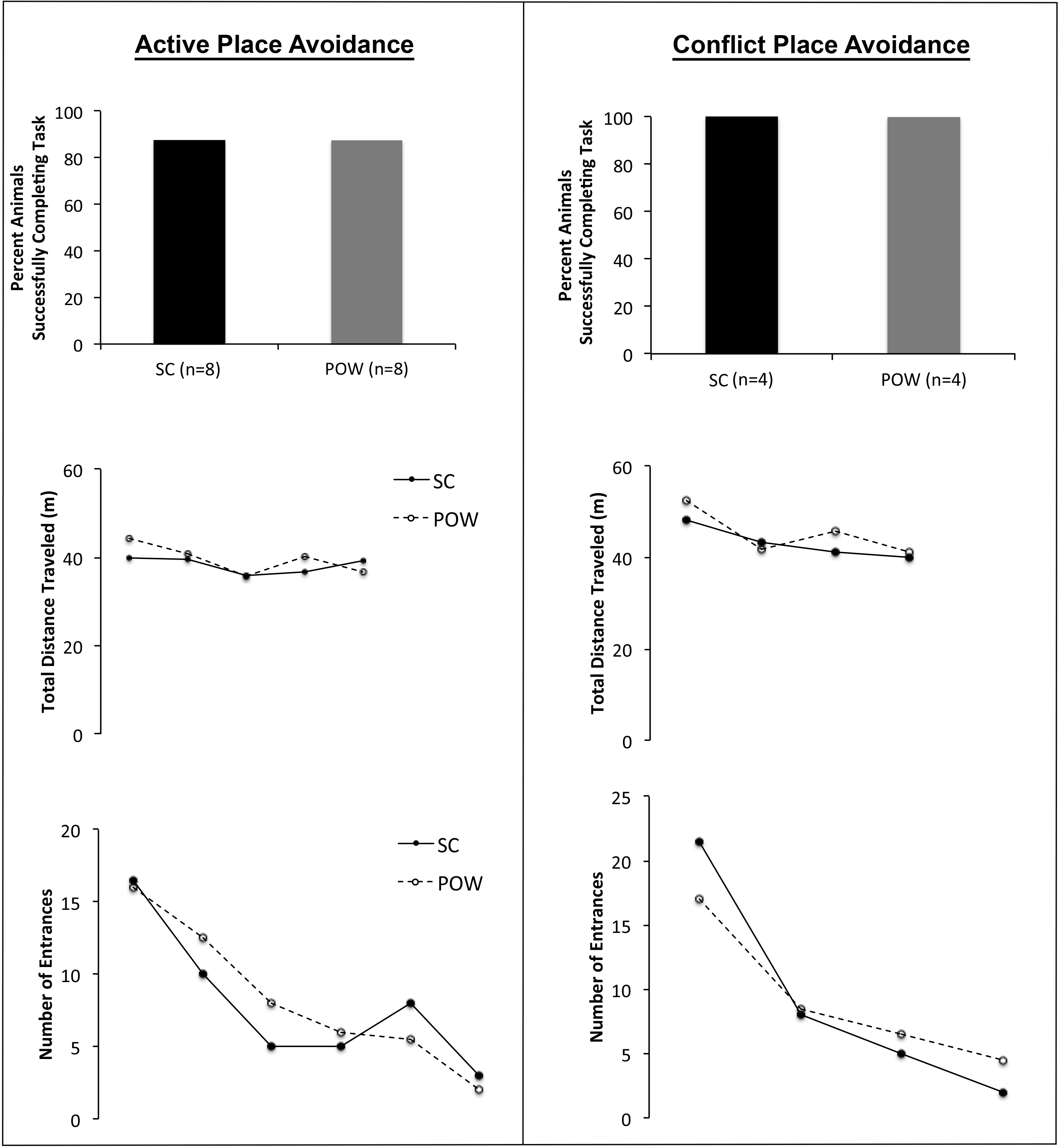

Supplement: S3 Fig — Rats exposed to FRα-Ab during the POW period successfully completed the active and conflict place avoidance tasks similarly to SC rats. They showed similar distance travelled as well as a decrease in entrances in subsequent trials, indicating successful learning of the task. (JPG) [file pone.0152249.s009.jpg]
